# Cd_3_P_2_/Zn_3_P_2_ Core-Shell Nanocrystals: Synthesis and Optical Properties

**DOI:** 10.3390/nano12193364

**Published:** 2022-09-27

**Authors:** Benjamin F. P. McVey, Robert A. Swain, Delphine Lagarde, Wilfried-Solo Ojo, Kaltoum Bakkouche, Cécile Marcelot, Bénédicte Warot, Yann Tison, Hervé Martinez, Bruno Chaudret, Céline Nayral, Fabien Delpech

**Affiliations:** 1 LPCNO, Université de Toulouse, CNRS, INSA, UPS, 135 Avenue de Rangueil, 31077 Toulouse, France; 2 Euromed Research Center, Engineering Division, Euro-Med University of Fez (UEMF), Route de Meknes, Rond-Point de Bensouda, Fès 30070, Morocco; 3 CEMES CNRS UPR 8011 and Université de Toulouse, 29 rue Jeanne Marvig, BP 94347, CEDEX 4, 31055 Toulouse, France; 4 Universite de Pau et des Pays de l’Adour, E2S UPPA, CNRS UMR 5254, IPREM, 64053 Pau, France; Electrochemical Energy Storage Network (RS2E), CNRS FR3459, 33 Rue Saint Leu, CEDEX, 80039 Amiens, France; 5 Centrale Casablanca, Centre de Recherche Systèmes Complexes et Interaction, Bouskoura 27182, Morocco

**Keywords:** nanomaterials, quantum dots, nanocrystal, core-shell nanostructure, cadmium phosphide, zinc phosphide, synthesis, shelling

## Abstract

II–V semiconductor nanocrystals such as Cd_3_P_2_ and Zn_3_P_2_ have enormous potential as materials in next-generation optoelectronic devices requiring active optical properties across the visible and infrared range. To date, this potential has been unfulfilled due to their inherent instability with respect to air and moisture. Core-shell system Cd_3_P_2_/Zn_3_P_2_ is synthesized and studied from structural (morphology, crystallinity, shell diameter), chemical (composition of core, shell, and ligand sphere), and optical perspectives (absorbance, emission-steady state and time resolved, quantum yield, and air stability). The improvements achieved by coating with Zn_3_P_2_ are likely due to its identical crystal structure to Cd_3_P_2_ (tetragonal), highlighting the key role crystallographic concerns play in creating cutting edge core-shell NCs.

## 1. Introduction

The size, surface, and composition-dependent optical properties of semiconductor nanocrystals (NCs) give them great potential in range of biomedical (imaging, sensing) and optoelectronic (LEDs, solar cells) applications [1,2,3,4,5]. As synthesized, the optical properties of semiconductor NCs are typically sensitive to ambient conditions (air, moisture), so to truly exploit them in next-generation biomedical and optoelectronic applications, surface passivation reactions are required [1]. The formation of a core-shell semiconductor NC is the most common surface passivation reaction, involving the growth of another material, usually a semiconductor over the NC core. The optical properties of a core-shell NC are highly tunable by considering the electronic properties of the core and shell materials [6,7,8]. For example, by changing the band alignments of the core and shell, a whole host of optical properties can be altered including absorbance and emission maximum, quantum yield, blinking rates, and an increased resistance to oxidation can be gained [9,10,11,12]. There are countless examples of systems, II–VI, IV–VI, and III–V-based nanomaterials where the optical properties have been transformed and exploited thanks to the development of robust core-shell syntheses [10,13,14].

The II–V semiconductors, Zn_3_P_2_, Cd_3_P_2_, Zn_3_As_2_, Cd_3_As_2_, and their alloys represent an emerging class of semiconductor NCs [15,16,17,18,19] with promise in light harvesting and detection applications. Surface passivation reactions for II–V are underdeveloped compared to the conventional II–VI and IV–VI materials with only a handful of successful reports [20,21,22]. Cd_3_P_2_ absorbs light on a large wavelengths scale with emission across the visible and NIR range (450–1500 nm) with quantum yields of up to 70%, and consequently is an ideal material for light energy conversion, optoelectronic, and telecommunication applications [23,24,25,26,27]. With robust formation protocols [26,27,28,29,30], Cd_3_P_2_ NCs appear as a perfect model to take up the challenges associated to this family and in particular that of growing a shell. Indeed, one of the biggest challenges in utilizing the II–V semiconductors is their extreme sensitivity to air and moisture. To the best of our knowledge, only two examples of coating have been reported for Cd_3_P_2_. Our group synthesized Cd_3_P_2_/ZnS core-shell NCs which showed stable emission 6 weeks post air exposure [26]. However, the shell was thin (0.3 nm), leading to batch dependent air stability. Ding et al. [31] reported the encapsulation of Cd_3_P_2_ NCs within silica nanobeads and polystyrene microspheres. Multiple QDs are contained within the microspheres making their practicality problematic [31]. These strategies having their limits, moving forward there is a great opportunity to study shelling methods for II–V semiconductor nanocrystals.

The purpose of this paper is to investigate the shelling of Cd_3_P_2_ nanocrystals with Zn_3_P_2_ which is an alternate material exhibiting tetragonal structure (this system could be seen as the analog of CdSe/ZnS). The synthesis and optical properties of core/shell Cd_3_P_2_/Zn_3_P_2_ NCs are comprehensively examined from chemical, structural, and optical perspectives. In contrast to the cubic ZnS material previously used, Zn_3_P_2_ leads to a robust type I heterostructure and opens new avenues of fundamental study and Cd_3_P_2_ based applications.

## 2. Materials and Methods

### 2.1. General Synthetic Considerations

All synthetic modifications were carried out in either an argon-filled glovebox with O_2_ and H_2_O levels below 0.1 ppm or an argon-filled Schlenk line with a vacuum level between 100 and 150 mTorr. All used glassware was dried in an oven (115 °C) overnight before use. Solvents used, mesitylene, toluene, and pentane, were collected from a puresolv solvent system and degassed with argon for at least 1 h before loading them into the glovebox. Oleylamine was degassed with argon and dried over 4 Å molecular sieves before use.

### 2.2. Synthesis and Purification of Cd_3_P_2_ Cores Stabilized with Hexadecylamine

*In situ synthesis of Cd(OAc)_2_(HDA)_2_*: In an argon-filled glovebox 0.1728 g (0.75 mmol) of anhydrous cadmium (II) acetate (99% STREM, Newburyport, MA, USA) is added to a 50 mL Schlenk tube along with either 0.4221 g (1.75 mmol) of hexadecylamine (HDA) (98% Sigma Aldrich, St. Louis, MO, USA). Next, 4 mL of anhydrous toluene (99.9% Honeywell, Charlotte, NC, USA) is added to Schlenk tube, which is then sealed via septa (mixture appears cloudy). The Schlenk tube is then brought out of the glovebox and hooked up to a Schlenk line. The rubber tube connecting the Schlenk tube to the Schlenk line then undergoes 3 cycles of evacuation and purging with argon, before opening the Schlenk tube to the line. The Schlenk tube is then plunged into an oil bath set to 40 °C. The mixture is left to react for 3 h, to insure precursor formation.

*Synthesis of Cd_3_P_2_ NCs*: In an argon-filled glovebox, 0.0671 g (0.25 mmol) of tris(trimethylsilyl) phosphine (98% STREM) is weighed out and dispersed in 1 mL of toluene. **CAUTION: tris(trimethylsilyl) phosphine is pyrophoric, producing toxic byproducts upon exposure to air and moisture.** Tris(trimethylsilyl) phosphine is then withdrawn into a 3 mL syringe and brought out of the glovebox. The syringe containing tris(trimethylsilyl) phosphine is then inserted into the Schlenk tube containing Cd(OAc)_2_(HDA)_2_ and is swiftly injected. Immediately upon injection, a color change is observed from cloudy white (Cd(OAc)_2_(HDA)_2_) to yellow. Over the next 5 min, several more color changes are observed, going from yellow to orange, then red, and finally dark red. After 2–3 h, the color resembles a dark velvet green. The reaction is then left to react overnight (18 h).

*Purification of Cd_3_P_2_ cores stabilized with hexadecylamine:* Unpurified Cd_3_P_2_ cores are then transferred into a glovebox and added to a 25 mL Nalgene centrifuge tube (Thermo Fisher) that contains a gas tight rubber O-ring (ensuring an argon atmosphere is maintained during centrifugation). Anhydrous acetonitrile (99.8% Sigma Aldrich) is then added to the unpurified Cd_3_P_2_ cores in a 3:1 ratio (i.e., 12 mL of acetonitrile to 4 mL of Cd_3_P_2_ cores). The centrifuge tube is then spun at 25,000 RPM for 25 min. After centrifugation, the centrifuge tube is re-entered into the glovebox and the supernatant (light yellow color) is decanted. The centrifuge tube containing Cd_3_P_2_ as a pellet is then dried under vacuum on a Schlenk line to remove traces of toluene and acetonitrile. Washed Cd_3_P_2_ cores are then dispersed in 5 mL of oleylamine (98% primary amine Sigma Aldrich).

### 2.3. Synthesis and Purification of Cd_3_P_2_/Zn_3_P_2_ Core-Shell Nanocrystals

*Synthesis of Cd_3_P_2_/Zn_3_P_2_*: 2 mL (0.054 mmol) of purified Cd_3_P_2_ cores stabilized with hexadecylamine is added to a 50 mL RB flask. The RB flask is then brought out of the glove box and connected to a Schlenk line. After 3 cycles of evacuation and purging with argon, the RB flask is opened to the line. A temperature probe is then put through one of the RB flasks septa and the reaction heated to 160 °C (ramp rate 25 °C per minute). When the reaction reaches 160 °C, 0.112 g (0.324 mmol) of zinc (II) tris(N,N′-diisopropylacetamidinato) dispersed in 1 mL of oleylamine is added dropwise over a one minute period. After 2 min, 0.0054 g (0.216 mmol) of tris(trimethylsilyl) phosphine (98% STREM) dispersed in 1 mL of oleylamine is added dropwise to the RB flask over a one-minute period. The reaction mixture is then heated to 180 ° C and left for 2 h.

*Purification of Cd_3_P_2_/Zn_3_P_2_:* Cd_3_P_2_/Zn_3_P_2_ synthesized at low and high temperature is purified in an identical fashion to Cd_3_P_2_ cores. The only difference is the purification cycles are repeated twice more with Cd_3_P_2_/Zn_3_P_2_, i.e., giving a total of 3 purification cycles. Post purification Cd_3_P_2_/Zn_3_P_2_ are dispersed in toluene.

### 2.4. Sample Characterization

Low- and high-resolution TEM analysis is performed at the “Centre De Microcaractérisation Raimond Castaing” on a JEOL JEM 1400 electron microscope operating at 120 kV with a point resolution of 4.5 Å and for HRTEM analysis on a JEOL 2100F operating at 200kV with a point resolution of 2.5 Å. Additional high resolution TEM is performed in a Hitachi HF3300 microscope equipped with a Cs corrector. STEM-EDS is performed in a Phillips CM20FEG equipped with a Brücker SDD detector. Samples for TEM analysis are prepared in glovebox by slow evaporation of a drop of the colloidal solution deposited onto a carbon-covered copper grid. The size distributions are determined by measuring at least 250 particles using image J software.

X-ray diffraction spectra are recorded on an MPD Pro Panalytical spectrometer using Co Kα radiation with a Kapton film holder. Before sample preparation, a small ring of grease is drawn on the Kapton film. For sample preparation, a highly concentrated solution of nanocrystals dispersed in pentane is drop cast onto Kapton foil within the previously mentioned grease ring. When nanocrystal film is dry, a second piece of Kapton film is placed on top. This second piece becomes stuck to the first piece due to the presence of the grease ring. The sealed Kapton films are then placed in a holder and brought out of the glovebox for XRD analysis.

Solid-state NMR experiments are recorded on a Bruker Avance III 400 spectrometer. Samples are packed into 3.2 mm zirconia rotors inside an argon-filled glovebox. The rotors are spun between 8 to 18 kHz at room temperature. For 1H MAS and 13C DP (direct polarization) MAS experiments, a small flip angle (~30°) is used with recycle delays of 5 s and 10 s, respectively. ^31^P Hahn-echo is performed with a recycle delay of 60 s and 6 s, respectively. ^13^C CP-MAS and ^31^P CP-MAS spectra are recorded with a recycle delay of 2 s and contact times of 2 ms, 2 ms and 3 ms, respectively. All chemical shifts for ^1^H and ^13^C are relative to TMS. ^31^P chemical shifts are referenced to an external 85% H_3_PO_4_ sample.

XPS measurements are recorded on a Thermo Electron Kα spectrometer with a base pressure of 5 × 10^−9^ mbars. For high-resolution spectra, a pass energy of 20 eV is used and charging effect is neutralized with a dual e^−^/Ar^+^ source. The C1s core peak of adventitious carbon at 285.0 eV is used as an internal energy reference. The data are treated with Casaxps using Gaussian–Lorentzian combinations (GL30) for the fits and Scofield photoionization cross-section [32], corrected for the analysis depth and the analyzer’s transmission function, for quantification.

UV–Visible Absorbance spectra are measured by using a Perkin Elmer lambda 35 scanning spectrometer with the sample in a 2 mm cells. Emission spectra are recorded with excitation laser centered at 402 nm (Nichia-NLVH 3000E). The light is then dispersed by a monochromator PI Acton SpectraPro 2500i. The detection is done 90° by CDD camera (Spec-10:100 BR/LN). Acquisition is performed by Winspec32 software.

For determinations of quantum yield (QY), the comparative method outlined by Williams et al. is used. The internal standard used is rhodamine 6G.

For time resolved emission measurements (TR emission) experiments are performed using 1.5 ps laser pulses generated by a tunable mode-locked Ti:Sa laser. A pulse picker is used to reduce the repetition rate from 80 MHz to 0.04 MHz and a second harmonic generation unit converted the excitation wavelength down to 390 nm. The laser beam is focused onto the cuvette on a 1/e diameter spot of ~100 µm and an average power of 50 µW. The PL signal is dispersed by a f#-6.5 spectrometer and detected as a function of time by a synchro-scan Hamamatsu streak camera with an overall temporal resolution of 35 ns.

## 3. Results and Discussion

Cd_3_P_2_ NCs are prepared using our already published protocol based on Cd(OAc)_2_(OcAm)_2_ (OcAm = octylamine) and tris(trimethylsilyl) phosphine (PTMS) as reagents [26]. Cd_3_P_2_ cores synthetized with octylamine cannot be precipitated via centrifugation so hexadecylamine is substituted for octylamine as the surfactant. Figure S1 shows a TEM image of these synthesized Cd_3_P_2_ cores which are roughly spherical in shape and monodisperse with average size of 3.6 ± 0.4 nm (σ = 11%) matching well with previous reports [26].

**Scheme 1:**
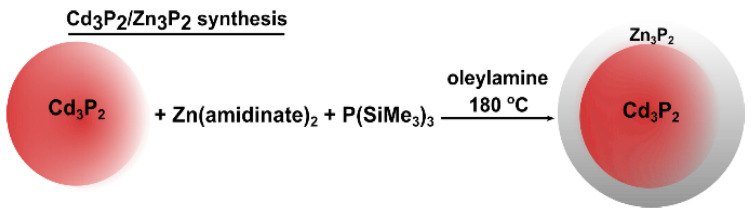
Scheme detailing the synthesis of a Zn_3_P_2_ shell on top of Cd_3_P_2_ cores.

**Figure 1:**
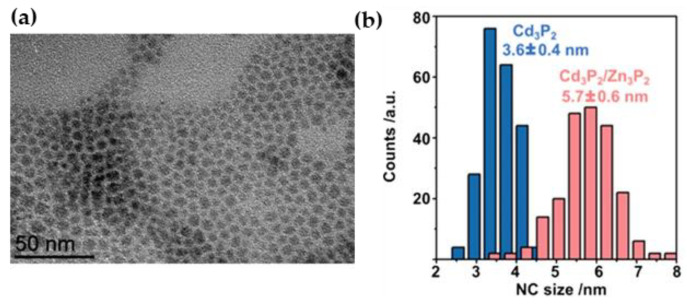
(**a**) TEM image of Cd_3_P_2_/Zn_3_P_2_ core-shell NCs. (**b**) Histograms of measured NC sizes for Cd_3_P_2_ cores and Cd_3_P_2_/Zn_3_P_2_ core-shell NCs.

Our Zn_3_P_2_ shelling protocol (Scheme 1) relies on the use of novel zinc precursor bis-diisopropylacetamidinato zinc(II), referred to as zinc(II) amidinate. This complex is selected for its high reactivity which proved to be highly relevant for the passivation of InP QDs and for the synthesis of Zn_3_P_2_ NCs [33]. Briefly, purified Cd_3_P_2_ cores are combined in a round bottom (RB) flask with Zn(amidinate)_2_ and oleylamine as the solvent. The RB flask is then heated to 180°C, at temperature, P(SiMe_3_)_3_ dissolved in oleylamine is slowly added dropwise. Key to the syntheses’ success is the removal of excess Cd precursor from the Cd_3_P_2_ cores, via centrifugation, before shelling. If Cd_3_P_2_ cores are used as synthesized, the excess Cd precursor reacts with P(SiMe_3_)_3_ preferentially, leading to the formation of additional Cd_3_P_2_ NCs. Figure 1a shows a TEM image of Cd_3_P_2_/Zn_3_P_2_ NCs grown from Cd_3_P_2_ cores stabilized with hexadecylamine. The shape of Cd_3_P_2_/Zn_3_P_2_ NCs is uniformly spherical in nature, similar to the Cd_3_P_2_ cores. The average size of Cd_3_P_2_/Zn_3_P_2_ NCs is 5.7 ± 0.6 nm (σ = 11%) with a shell width of ca 1.0 nm (Figure 1b). The composition of these shelled NCs is determined using ICP-MS, EDX, and XPS and all these techniques give consistent results close to Cd_3_P_2_/Zn_3_P_2_ (Table S1).

The XRD pattern of Cd_3_P_2_ cores and Cd_3_P_2_/Zn_3_P_2_ are seen in Figure S2. The XRD pattern of Cd_3_P_2_/Zn_3_P_2_ shows a peak at 50.7° matching the (220) facet of Cd_3_P_2_ (cubic or tetragonal structures are not distinguishable at these sizes of crystallites) having shifted towards higher diffraction angles post shelling, consistent with core-shell formation (lattice constraint as usually described) [6]. The XRD peak of Cd_3_P_2_/Zn_3_P_2_ is also sharper as evidenced by a decrease in the peak FWHM (Table S2) and HRTEM of Cd_3_P_2_/Zn_3_P_2_ (seen in Figure S2) shows the good crystallinity of the particle.

To examine the coordination sphere of Cd_3_P_2_/Zn_3_P_2_, ^1^H-^13^C cross polarization (CP) MAS NMR is used. The CP spectrum of Cd_3_P_2_/Zn_3_P_2_ shows characteristic peaks at 129.6 and 41.4 ppm matching the carbons double bond and the α-CH_2_ resonances of oleylamine, respectively (Figure S3a), demonstrating that the amine is stabilizing the NCs. The P environment of Cd_3_P_2_/Zn_3_P_2_ is examined using Hahn-echo ^31^P MAS NMR (Figure S3b). This spectrum shows P signal post shelling with one broad peak centralized around −225 ppm and a sharp peak around 6 ppm matching well with metal phosphides and oxidized phosphorus. Integrating the peak area for metal phosphide and oxidized phosphorus gives an oxidation level of 15%. The source of oxidation likely comes from the presence of the acetate ligand in the Cd precursor, similar to previous reports on InP oxidation with In(OAc)_3_ precursors [34,35].

XPS analysis is also used to characterize Cd_3_P_2_/Zn_3_P_2_ (Figure S4). The presence of Cd, P, and Zn was detected. The P 2p region exhibits 2 doublets. A contribution at low binding energy, 128.1–129.0 eV, is assigned to Cd-P/Zn-P bonding within the nanoparticles [36]. The binding energy of the second doublet, found at 132.8–133.7 eV, is close to the values reported for phosphorus in nitrogen or oxygen environment. Therefore, consistently with the NMR conclusions, this second doublet can be attributed to the phosphate P atoms located at the surface. Regarding cadmium, the Cd 3d doublet is found at 404.9–411.6 eV, which is consistent with a phosphorus environment. The Zn 2p region displays 1 doublet at 1022.0–1045.0 eV which is also in good agreement with Zn-P bonding [37].

Overall, the Zn_3_P_2_ shell shows high shape uniformity and the improvement in shell thickness (compared to our previous work on ZnS shelling) is likely due to Cd_3_P_2_ and Zn_3_P_2_ both having a more favorable lattice mismatch. Due to the small size of crystallites and the low imaging contrast on metal phosphides, XRD and HRTEM on our Cd_3_P_2_ samples do not allow to discriminate cubic or tetragonal Cd_3_P_2_ structures. However, regarding the easy growth of Zn_3_P_2_ (tetragonal structure) shell compared to ZnS (cubic structure) shell on our Cd_3_P_2_ cores, it is more likely that they exhibit a tetragonal crystal structure as the shelling material. This is consistent with a previous work of Bawendi which showed the possibility to grow a shell of Cd_3_P_2_ on tetragonal Cd_3_As_2_ QDs [18]. Interestingly, Cossairt succeeded in growing CdS (cubic structure) tetrapods on cubic Cd_3_P_2_ seeds [29]. This is highlighting, once again, the important role that crystallographic matching plays on successful growth of heterostructures, and in our case, in the growth of Zn_3_P_2_ shell with high uniformity and robustness.

**Figure 2:**
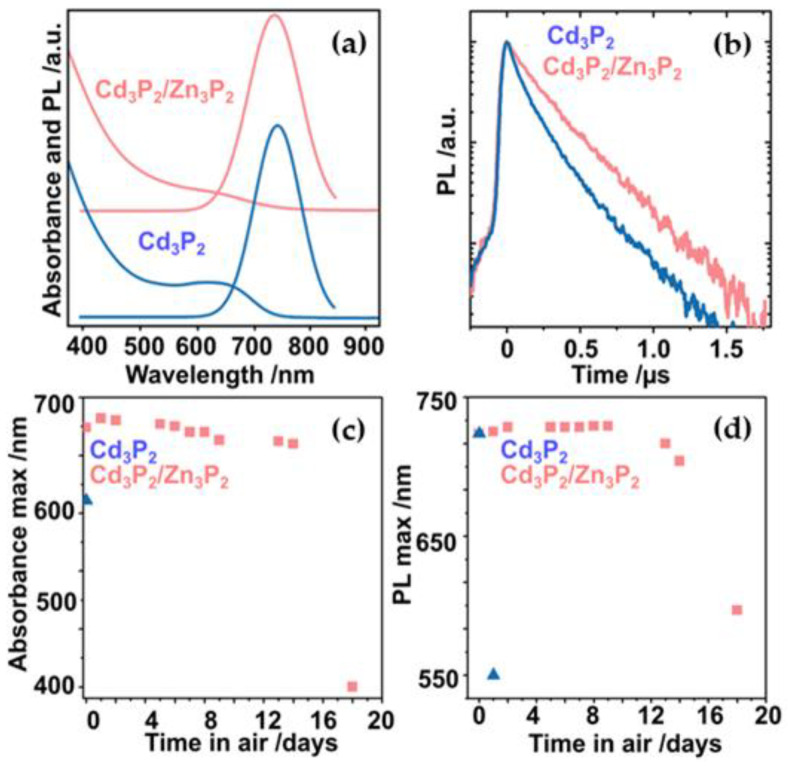
(**a**) Absorbance and emission of Cd_3_P_2_ cores (blue) and Cd_3_P_2_/Zn_3_P_2_ (red) core-shell NCs sealed in gas tight cuvettes under an Ar atmosphere. (**b**) Time-resolved emission spectra of Cd_3_P_2_ cores (blue) and Cd_3_P_2_/Zn_3_P_2_ (red) core-shell NCs (**c**) and (**d**) graph showing changes in absorbance and emission maxima of Cd_3_P_2_ (blue triangles) and Cd_3_P_2_/Zn_3_P_2_ (red squares) NCs upon exposure to air over a 16-day period.

The optical properties of Cd_3_P_2_ and Cd_3_P_2_/Zn_3_P_2_ are characterized using absorbance and emission (steady state and time resolved) spectroscopies. The absorbance and emission spectra of Cd_3_P_2_ cores (blue) and Cd_3_P_2_/Zn_3_P_2_ (red) core-shell QDs are seen in Figure 2a. The absorbance spectra of Cd_3_P_2_ cores demonstrates a broad hump from its onset point, 710 nm, with an absorption maximum of 644nm, matching well with previous reports on this particle size [26]. For Cd_3_P_2_/Zn_3_P_2_ NCs, the absorbance spectrum is similarly broad from its onset, 720 nm, with an absorbance maximum of 649 nm. The emission spectra of Cd_3_P_2_ and Cd_3_P_2_/Zn_3_P_2_ NCs taken at an excitation wavelength of 430 nm are also seen in Figure 2a. Cd_3_P_2_ cores demonstrate an emission maximum of 729 nm with a full width at half maximum (FWHM) of 80 nm, consistently with previous reports [26]. Cd_3_P_2_/Zn_3_P_2_ core-shells have an emission maximum of 736 nm with a FWHM of 90 nm, giving a 7 nm redshift relative to Cd_3_P_2_ cores. These observations are consistent with previous reports for CdSe/ZnS [10] or the related Cd_3_P_2_/ZnS QDs [26] can be assigned to the leakage of the exciton into the shell material.

The higher bandgaps of Zn_3_P_2_ (1.5 eV) compared to Cd_3_P_2_ (0.5 eV) should lead to similar absorbance and emission maxima post-shelling due to their type I band alignment [6]. The minor absorbance and emission redshifts (5–10 nm) observed for Cd_3_P_2_/Zn_3_P_2_ are consistent with a slight overlap of the exciton into the shell as described by previous reports of type I core-shell QDs (CdSe/ZnS, InP/ZnSe) [10,38].

The QYs of Cd_3_P_2_ and Cd_3_P_2_/Zn_3_P_2_ NCs are calculated by the comparative method using rhodamine 6G as a reference. The QYs of Cd_3_P_2_ and Cd_3_P_2_/Zn_3_P_2_ NCs are, respectively, 52% and 45%. As this slight difference is within the uncertainty of the comparative method, no significant modification of the QY can be observed post-shelling. This result is reminiscent to our previous work on ZnS coating [26], where the QY of the Cd_3_P_2_ cores (around 50%) is not increased or impacted by the presence of a layer of ZnS.

To gain insight into the fundamental optical properties of Cd_3_P_2_/Zn_3_P_2_ NCs, time resolved emission spectroscopy is used. All NCs require biexponential fits, with time constants summarized in Table S3. Figure 2b shows the fitted time resolved emission data of Cd_3_P_2_/Zn_3_P_2_ and Cd_3_P_2_ NCs for reference. The two emission components of Cd_3_P_2_ have lifetimes of 110 ns (τ1) and 290 ns (τ2) with contributions of 75% and 25%, respectively (average 155 ns). These τ1 and τ2 values are consistent with an emission from two different types of defects, consistently with previous results for both NCs and bulk Cd_3_P_2_ [30]. After shelling, the two emission components of Cd_3_P_2_/Zn_3_P_2_ have lifetimes of 140 ns and 290 ns, with contributions of 25% and 75%, respectively (average 253 ns). The difference between the lifetimes before and after shelling is not significant. However, after shelling, the contribution from the first decay component has decreased drastically, 75% to 25%. We assume that the shelling process has led to a partial passivation of the states responsible of the τ1 component. The chemical nature of these optically active defects might be related to the surface oxidized phosphorus species, PO_x_, as seen in the ^31^P NMR. Similar observations are seen in the optical properties of InP/ZnX (X = S, Se) [39], highlighting the detrimental nature oxides can play on NC optical properties. However, compared to well-studied materials such as CdSe, the band structure of both Cd_3_P_2_ and Zn_3_P_2_ is not well understood [27,40]. Long-lived emissive transitions in Cd_3_P_2_ QDs have been described in the literature but their cause is still unknown [40]. In our case, the second long-lived transition is not altered by the presence of the Zn_3_P_2_ shell, its origin could be an intraband transition within the Cd_3_P_2_ bandgap, and therefore from an energy perspective, it is unaffected by the higher bandgap Zn_3_P_2_ shell. To fully understand the origin of these emissive transitions, more research is needed.

The stability of Cd_3_P_2_ and Cd_3_P_2_/Zn_3_P_2_ optical properties with respect to air was monitored using absorbance and emission spectroscopy. Figure 2c,d show respectively the absorbance and the emission maxima of Cd_3_P_2_ and Cd_3_P_2_/Zn_3_P_2_ exposed to air over an 18-day period. After 1 day of air exposure, the absorbance and the emission of Cd_3_P_2_ NCs strongly blueshift, demonstrating the high sensitivity of Cd_3_P_2_ cores to air. In contrast, the presence of a Zn_3_P_2_ shell clearly improves the optical stability of Cd_3_P_2_ as reliable QD absorbance and emission remains essentially unchanged for up to 14 days post exposure to air. In comparison, assembly of Cd_3_P_2_ QDs embedded in silica, showed a stability of only 2.5 days in buffer solution [31].

In summary, looking for new directions towards the successful shelling for Cd_3_P_2_ and potentially other II–V materials, Zn_3_P_2_ was explored for the first time as a shelling material. A new synthetic procedure was developed through the use of Zn(amidinate)_2_ and P(SiMe_3_)_3_ as zinc and phosphorus precursors, respectively. Low/high resolution TEM in conjunction with XRD measurements demonstrated that Zn_3_P_2_ shells could be easily grown onto Cd_3_P_2_ cores. Cd_3_P_2_/Zn_3_P_2_ core-shell NCs demonstrated nearly identical absorbance/emission maxima and QY compared to Cd_3_P_2_ core. Importantly, the Cd_3_P_2_/Zn_3_P_2_ core-shell NCs had significantly higher air stability demonstrating stable QD absorbance and emission up to 14 days post air exposure, compared to Cd_3_P_2_ cores which lost both 1 day post air exposure. This work represents thus a required step towards their practical use in a range of visible/IR based optoelectronic devices.

For the large majority of applications where semiconductors NCs are currently utilized, their true exploitation began after the development of robust shelling materials and strategies. Zn_3_P_2_ exhibiting a tetragonal structure and a bandgap (1.5 eV) higher than the majority of II–V materials, the shelling developed here should be broadly applicable to other II–V tetragonal QDs (Cd_3_As_2_, Cd_x_Zn_y_As_2_) analogous to shelling common place II–VI and III–V cubic QDs (CdSe, CdS, InP) with ZnS. With this synthesis, the next generation of II–V materials can be unlocked for exploration, from fundamental optics to applied materials.

## Data Availability

Not applicable.

## Supplementary Materials

The following supporting information can be downloaded at: https://www.mdpi.com/article/10.3390/nano12193364/s1, Figure S1: Low (left) and high (right) resolution transmission electron microscopy images of Cd_3_P_2_ cores synthesized with hexadecylamine as the surfactant; Figure S2: High resolution transmission electron microscopy (a) of Cd_3_P_2_/Zn_3_P_2_ nanocrystals and XRD patterns (b) of Cd_3_P_2_ cores (blue), Cd_3_P_2_/ Zn_3_P_2_ (red), the relevant reflections are highlighted for Cd_3_P_2_ 00-002-1182 and Zn_3_P_2_ 01-002-1264. The * symbol denotes reflections that are caused by the kapton film; Figure S3: Magic Angle Spinning NMR of Cd_3_P_2_/Zn_3_P_2_: (a) 13C CP MAS NMR spectra of Cd_3_P_2_/Zn_3_P_2_, (b) 31P Hahn-echo MAS NMR spectra of Cd_3_P_2_/Zn_3_P_2_; Figure S4: High resolution XPS of Cd_3_P_2_/Zn_3_P_2_: (a) P 2p and Zn 3s core peaks; (b) Cd 3d core peaks, the detection of a N 1s peak at ca. 399.0 eV is due to the presence of nitrogen atoms in the oleylamine ligands; (c) Zn 2p core peaks. The presence of carbon and oxygen is also detected through O 1s and C1 core peaks (not shown here); Table S1: Composition of synthesized nanocrystals from ICP-MS, EDX, and XPS measurements. XPS compositions were determined from the P 2p, Zn 3s and Cd 3d core peaks; Table S2: XRD peak maxima and full width at half maxima for the (220) plan of Cd_3_P_2_ and Cd_3_P_2_/Zn_3_P_2_; Table S3: Time-resolved emission measurements: average emission lifetimes of Cd_3_P_2_ and Cd_3_P_2_/Zn_3_P_2_ core-shell nanocrystals.

## References

[1] Boles M.A., Ling D., Hyeon T., Talapin D.V. (2016). The surface science of nanocrystals. Nat. Mater..

[2] Chen C., Corry B., Huang L., Hildebrandt N. (2019). In Situ Construction of a Cs_2_SnI_6_ Perovskite Nanocrystal/SnS2 Nanosheet Heterojunction with Boosted Interfacial Charge Transfer. J. Am. Chem. Soc..

[3] Cao F., Wang S., Wang F., Wu Q., Zhao D., Yang X. (2018). A Layer-by-Layer Growth Strategy for Large-Size InP/ZnSe/ZnS Core–Shell Quantum Dots Enabling High-Efficiency Light-Emitting Diodes. Chem. Mater..

[4] Wagner A.M., Knipe J.M., Orive G., Peppas N.A. (2019). Quantum dots in biomedical applications. Acta Biomater..

[5] Litvin A.P., Martynenko I.V., Purcell-Milton F., Baranov A.V., Fedorov A.V., Gun’ko Y.K. (2017). Colloidal quantum dots for optoelectronics. J. Mater. Chem. A.

[6] Reiss P., Protière M., Li L. (2009). Core/Shell Semiconductor Nanocrystals. Small.

[7] Liu H., Hao J., Li J., Cheng J., Gao Y., Lin X., Wang K., He T. (2020). Spectral and Nonlinear Optical Properties of Quasi-Type II CdSe/CdS Nanotadpoles. J. Phys. Chem. C.

[8] Gao Y., Qiu X., Zhao F., Xiao S., Li J., Lin X., Chen R., He T. (2020). Linear and nonlinear photophysical properties of ZnSe/CdS/ZnS core/shell/shell type II nanocrystals. Photonics Res..

[9] Ivanov S.A., Piryatinski A., Nanda J., Tretiak S., Zavadil K.R., Wallace W.O., Werder D., Klimov V.I. (2007). Type-II Core/Shell CdS/ZnSe Nanocrystals:  Synthesis, Electronic Structures, and Spectroscopic Properties. J. Am. Chem. Soc..

[10] Hines M.A., Guyot-Sionnest P. (1996). Synthesis and characterization of strongly luminescing ZnS-capped CdSe nanocrystals. J. Phys. Chem..

[11] Rabouw F.T., Antolinez F.V., Brechbühler R., Norris D.J. (2019). Microsecond blinking events in the fluorescence of colloidal quantum dots revealed by correlation analysis on preselected photons. J. Phys. Chem. Lett..

[12] Chen O., Zhao J., Chauhan V.P., Cui J., Wong C., Harris D.K., Wei H., Han H.-S., Fukumura D., Jain R.K. (2013). Compact high-quality CdSe-CdS core-shell nanocrystals with narrow emission linewidths and suppressed blinking. Nat. Mater..

[13] Brumer M., Kigel A., Amirav L., Sashchiuk A., Solomesch O., Tessler N., Lifshitz E. (2005). PbSe/PbS and PbSe/PbSexS1–x Core/Shell Nanocrystals. Adv. Funct. Mater..

[14] Kim Y., Ham S., Jang H., Min J.H., Chung H., Lee J., Kim D., Jang E. (2019). Bright and uniform green light emitting InP/ZnSe/ZnS quantum dots for wide color gamut displays. ACS Appl. Nano Mater..

[15] Glassy B.A., Cossairt B.M. (2017). II_3_V_2_ (II: Zn, Cd; V: P, As) Semiconductors: From Bulk Solids to Colloidal Nanocrystals. Small..

[16] Glassy B.A., Lai N.L., Cossairt B.M. (2017). Synthesis of Zn_3_As_2_ and (CdyZn1–y)_3_As_2_ Colloidal Quantum Dots. Chem. Mater..

[17] Glassy B.A., Cossairt B.M. (2015). Ternary synthesis of colloidal Zn_3_P_2_ quantum dots. Chem. Commun..

[18] Harris D.K., Allen P.M., Han H.-S., Walker B.J., Lee J., Bawendi M.G. (2011). Synthesis of Cadmium Arsenide Quantum Dots Luminescent in the Infrared. J. Am. Chem. Soc..

[19] Das A., Shamirian A., Snee P.T. (2016). Arsenic silylamide: An effective precursor for arsenide semiconductor nanocrystal synthesis. Chem. Mater..

[20] Luber E.J., Mobarok M.H., Buriak J.M. (2013). Solution-Processed Zinc Phosphide (α-Zn_3_P_2_) Colloidal Semiconducting Nanocrystals for Thin Film Photovoltaic Applications. ACS Nano..

[21] Miao S., Yang T., Hickey S.G., Lesnyak V., Rellinghaus B., Xu J., Eychmüller A. (2013). Emissive ZnO@Zn_3_P_2_ Nanocrystals: Synthesis, Optical, and Optoelectrochemical Properties. Small..

[22] Wadia C., Alivisatos A.P., Kammen D.M. (2009). Materials Availability Expands the Opportunity for Large-Scale Photovoltaics Deployment. Environ. Sci. Technol..

[23] Xie R., Zhang J., Zhao F., Yang W., Peng X. (2010). Synthesis of Monodisperse, Highly Emissive, and Size-Tunable Cd_3_P_2_ Nanocrystals. Chem. Mater..

[24] Miao S., Hickey S.G., Rellinghaus B., Waurisch C., Eychmüller A. (2010). Synthesis and Characterization of Cadmium Phosphide Quantum Dots Emitting in the Visible Red to Near-Infrared. J. Am. Chem. Soc..

[25] Miao S., Hickey S.G., Waurisch C., Lesnyak V., Otto T., Rellinghaus B., Eychmüller A. (2012). Synthesis of Monodisperse Cadmium Phosphide Nanoparticles Using ex-Situ Produced Phosphine. ACS Nano..

[26] Ojo W.-S., Xu S., Delpech F., Nayral C., Chaudret B. (2012). Room-Temperature Synthesis of Air-Stable and Size-Tunable Luminescent ZnS-Coated Cd_3_P_2_ Nanocrystals with High Quantum Yields. Angew. Chemie Int. Ed..

[27] Cao H., Liu Z., Zhu X., Peng J., Hu L., Xu S., Luo M., Ma W., Tang J., Liu H. (2015). PbS/Cd_3_P_2_ quantum heterojunction colloidal quantum dot solar cells. Nanotechnology..

[28] Mundy M.E., Ung D., Lai N.L., Jahrman E.P., Seidler G.T., Cossairt B.M. (2018). Aminophosphines as Versatile Precursors for the Synthesis of Metal Phosphide Nanocrystals. Chem. Mater..

[29] Enright M.J., Dou F.Y., Wu S., Rabe E.J., Monahan M., Friedfeld M.R., Schlenker C.W., Cossairt B.M. (2020). Seeded growth of nanoscale semiconductor tetrapods: Generality and the role of cation exchange. Chem. Mater..

[30] Kornowski A., Eichberger R., Giersig M., Weller H., Eychmüller A. (1996). Perspectives on the Physical Chemistry of Semiconductor Nanocrystals. J. Phys. Chem..

[31] Ding L., He S., Chen D., Huang M., Xu J., Hickey S.G., Eychmüller A., Yu S.-H., Miao S. (2014). Encapsulated Cd_3_P_2_ quantum dots emitting from the visible to the near infrared for bio-labelling applications. CrystEngComm.

[32] Scofield J.H. (1976). Hartree-Slater subshell photoionization cross-sections at 1254 and 1487 eV. J. Electron Spectrosc. Relat. Phenom..

[33] Swain R.A., McVey B.F.P., Virieux H., Ferrari F., Tison Y., Martinez H., Chaudret B., Nayral C., Delpech F. (2020). Sustainable quantum dot chemistry: Effects of precursor, solvent, and surface chemistry on the synthesis of Zn_3_P_2_ nanocrystals. Chem Comm..

[34] Cros-Gagneux A., Delpech F., Nayral C., Cornejo A., Coppel Y., Chaudret B. (2010). Surface chemistry of InP quantum dots: A comprehensive study. J. Am. Chem. Soc..

[35] Virieux H., le Troedec M., Cros-Gagneux A., Ojo W.-S., Delpech F., Nayral C., Martinez H., Chaudret B. (2012). InP/ZnS nanocrystals: Coupling NMR and XPS for fine surface and interface description. J. Am. Chem. Soc..

[36] Mobarok M.H., Luber E.J., Bernard G.M., Peng L., Wasylishen R.E., Buriak J.M. (2014). Phase-Pure Crystalline Zinc Phosphide Nanoparticles: Synthetic Approaches and Characterization. Chem. Mater..

[37] Huang K., Demadrille R., Silly M.G., Sirotti F., Reiss P., Renault O. (2010). Internal Structure of InP/ZnS Nanocrystals Unraveled by High-Resolution Soft X-ray Photoelectron Spectroscopy. ACS Nano.

[38] Dupont D., Tessier M.D., Smet P.F., Hens Z. (2017). Indium Phosphide-Based Quantum Dots with Shell-Enhanced Absorption for Luminescent Down-Conversion. Adv. Mater..

[39] Tessier M.D., Baquero E.A., Dupont D., Grigel V., Bladt E., Bals S., Coppel Y., Hens Z., Nayral C., Delpech F. (2018). Interfacial Oxidation and Photoluminescence of InP-Based Core/Shell Quantum Dots. Chem. Mater..

[40] Wu K., Liu Z., Zhu H., Lian T. (2013). Exciton Annihilation and Dissociation Dynamics in Group II–V Cd_3_P_2_ Quantum Dots. J. Phys. Chem. A.

