# Supplementary material for: Cd3P2/Zn3P2 Core-Shell Nanocrystals: Synthesis and Optical Properties
Source: Nanomaterials (Basel). 2022 Sep 27;12(19):3364. doi: 10.3390/nano12193364 (PMC9565233; doi:10.3390/nano12193364)

Supplementary Materials

Cd_3P_2/Zn_3P_2 Core-Shell Nanocrystals: Synthesis and Optical Properties

Benjamin F.P. McVey [1], Robert A. Swain [1], Delphine Lagarde [1], Wilfried-Solo Ojo [1], Kaltoum Bakkouche [1,2], Cécile Marcelot [3], Bénédicte Warot [3], Yann Tison [4], Hervé Martinez [4,5], Bruno Chaudret [1], Céline Nayral [1,*] and Fabien Delpech [1,*]

1 LPCNO, Université de Toulouse, CNRS, INSA, UPS, 135 Avenue de Rangueil, 31077 Toulouse, France; mcvey.bfp@gmail.com (B.F.P.M.); ras2250@columbia.edu (R.A.S.); delphine.lagarde@insa-toulouse.fr (D.L.); solowilly@yahoo.fr (W.-S.O.); bakkouch@insa-toulouse.fr (K.B.); chaudret@insa-toulouse.fr (B.C.)

2 Euromed Research Center, Engineering Division, Euro-Med University of Fez (UEMF), Route de Meknes, Rond-point de Bensouda, Fès 30070, Morocco

3 CEMES CNRS UPR 8011 and Université de Toulouse, 29 rue Jeanne Marvig, BP 94347, CEDEX 4, 31055 Toulouse, France; cecile.marcelot@cemes.fr (C.M.); benedicte.warot@cemes.fr (B.W.)

4 Universite de Pau et des Pays de l'Adour, E2S UPPA, CNRS UMR 5254, IPREM, 64053 Pau, France; Electrochemical Energy Storage Network (RS2E), CNRS FR3459, 33 Rue Saint Leu, CEDEX, 80039 Amiens, France; yann.tison@univ-pau.fr (Y.T.); herve.martinez@univ-pau.fr (H.M.)

5 Centrale Casablanca, Centre de Recherche Systèmes Complexes et Interaction, Bouskoura 27182, Morocco

* Correspondence: cnayral@insa-toulouse.fr (C.N.); fdelpech@insa-toulouse.fr (F.D.)

Figure S1: Low (left) and high (right) resolution transmission electron microscopy images of Cd_3P_2 cores synthesized with hexadecylamine as the surfactant.

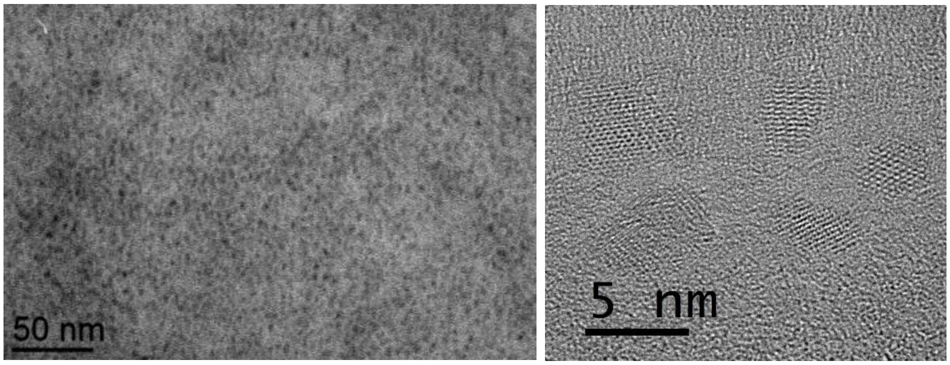

Figure S2: High resolution transmission electron microscopy of Cd_3P_2/Zn_3P_2 nanocrystals and XRD patterns of Cd_3P_2 cores (blue), Cd_3P_2/ Zn_3P_2 (red), the relevant reflections are highlighted for Cd_3P_2 00-002-1182 and Zn_3P_2 01-002-1264. The * symbol denotes reflections that are caused by the kapton film.

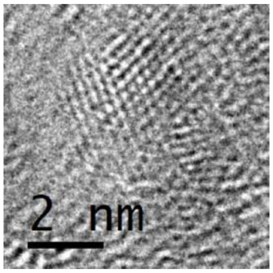

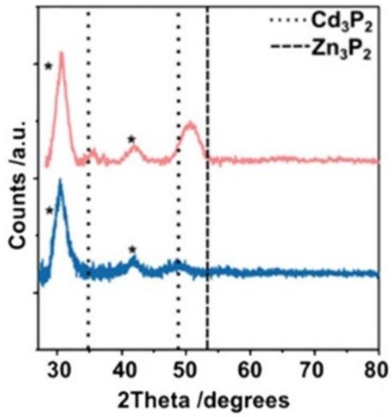

Table S1: Composition of synthesized nanocrystals from ICP-MS, EDX, and XPS measurements. XPS compositions were determined from the P 2p, Zn 3s and Cd 3d core peaks.

Sample	ICP-MS	EDX	XPS
Cd_3P_2	0.119 mmol Cd; 0.081 mmol P<br><br>($Cd_{2,94}P_2$)	64 % Cd; 36 % P<br><br>($Cd_{3.55}P_2$)	$Cd_{3.2}P_2$
Cd_3P_2/Zn_3P_2	0.111 mmol Cd; 0.322 mmol Zn; 0.273 mmol P<br><br>($Cd_{0,81}Zn_{2,36}P_2$)<br><br>With a $Cd_{2.94}P_2$ core, shell composition is found to be $Zn_{3.26}P_2$	18 % Cd; 45 % Zn; 37 % P<br><br>($Cd_{0,97}Zn_{2,43}P_2$)<br><br>With a $Cd_{3.55}P_2$ core, shell composition is found to be $Zn_{3,35}P_2$	$Cd_{1,1}Zn_{2,2}P_2$<br><br>With a $Cd_{3.2}P_2$ core, shell composition is found to be $Zn_{3,3}P_2$

Table S2: XRD peak maxima and Full Width at Half Maxima for the (220) plan of Cd_3P_2 and Cd_3P_2/Zn_3P_2

Sample	Peak maxima (°)	Peak FWHM (°)
Cd_3P_2 cores	48.8°	3.88
Cd_3P_2/Zn_3P_2	50.7°	2.95

Table S3: Time resolved emission measurements: average emission lifetimes of Cd_3P_2 and Cd_3P_2/Zn_3P_2 core-shell nanocrystals.

Sample	Coeff A1	Average lifetime (t_1)	Coeff A2	Average lifetime (t_2)
Cd_3P_2 cores	0,75	110 ns	0,25	290 ns
Cd_3P_2/Zn_3P_2	0,25	140 ns	0,75	290 ns

$I=A1*\exp(-t/t1)+A2*\exp(-t/t2)+yo$ (noise level)

Figure S3: Magic Angle Spinning NMR of Cd_3P_2/Zn_3P_2: 3a) 13C CP MAS NMR spectra of Cd_3P_2/Zn_3P_2, 3b) 31P Hahn-echo MAS NMR spectra of Cd_3P_2/Zn_3P_2.

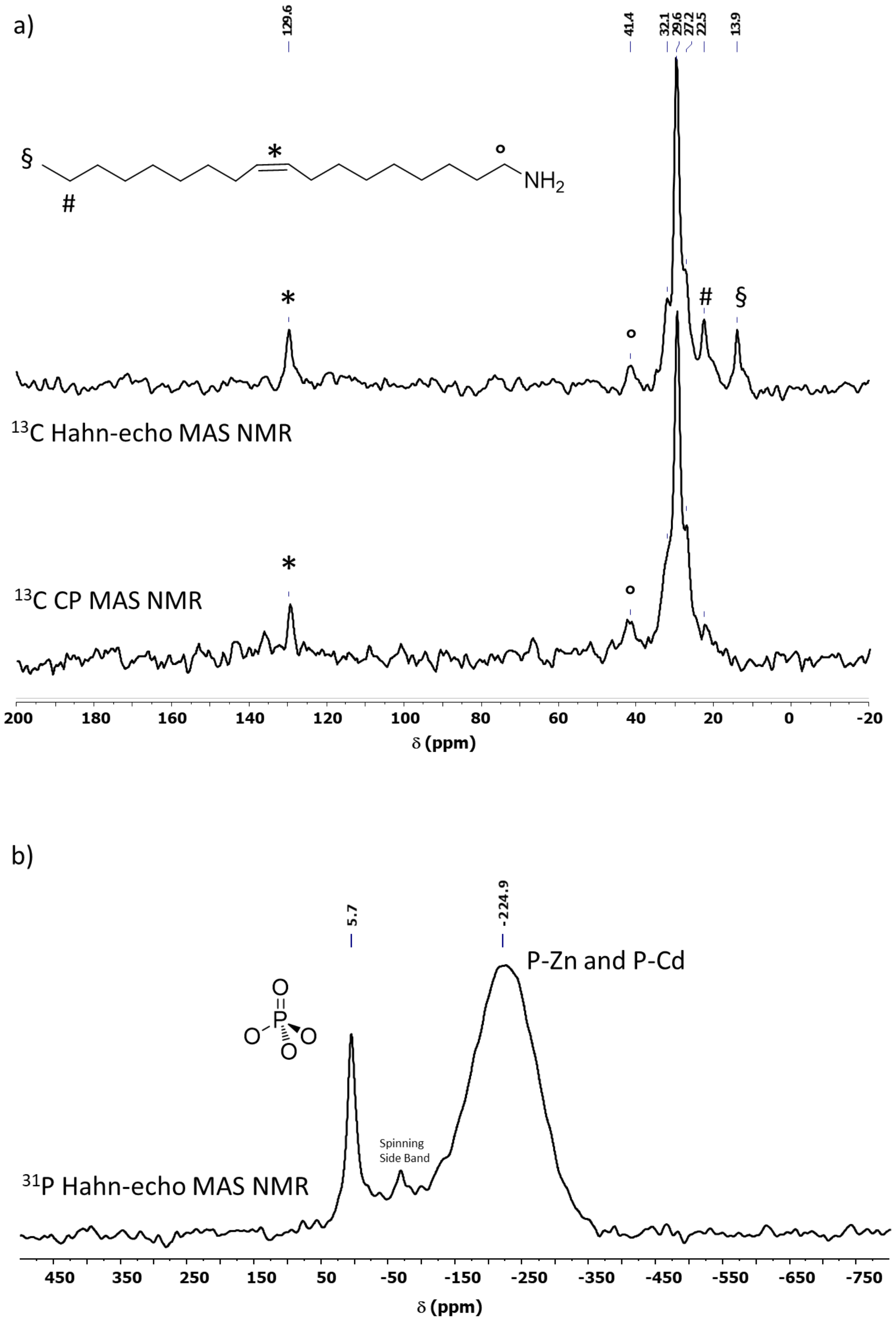

Figure S4: High resolution XPS of Cd_3P_2/Zn_3P_2: a) P 2p and Zn 3s core peaks; b) Cd 3d core peaks, the detection of a N 1s peak at ca. 399.0 eV is due to the presence of Nitrogen atoms in the oleylamine ligands; c) Zn 2p core peaks. The presence of Carbon and Oxygen is also detected through O 1s and C1 core peaks (not shown here).

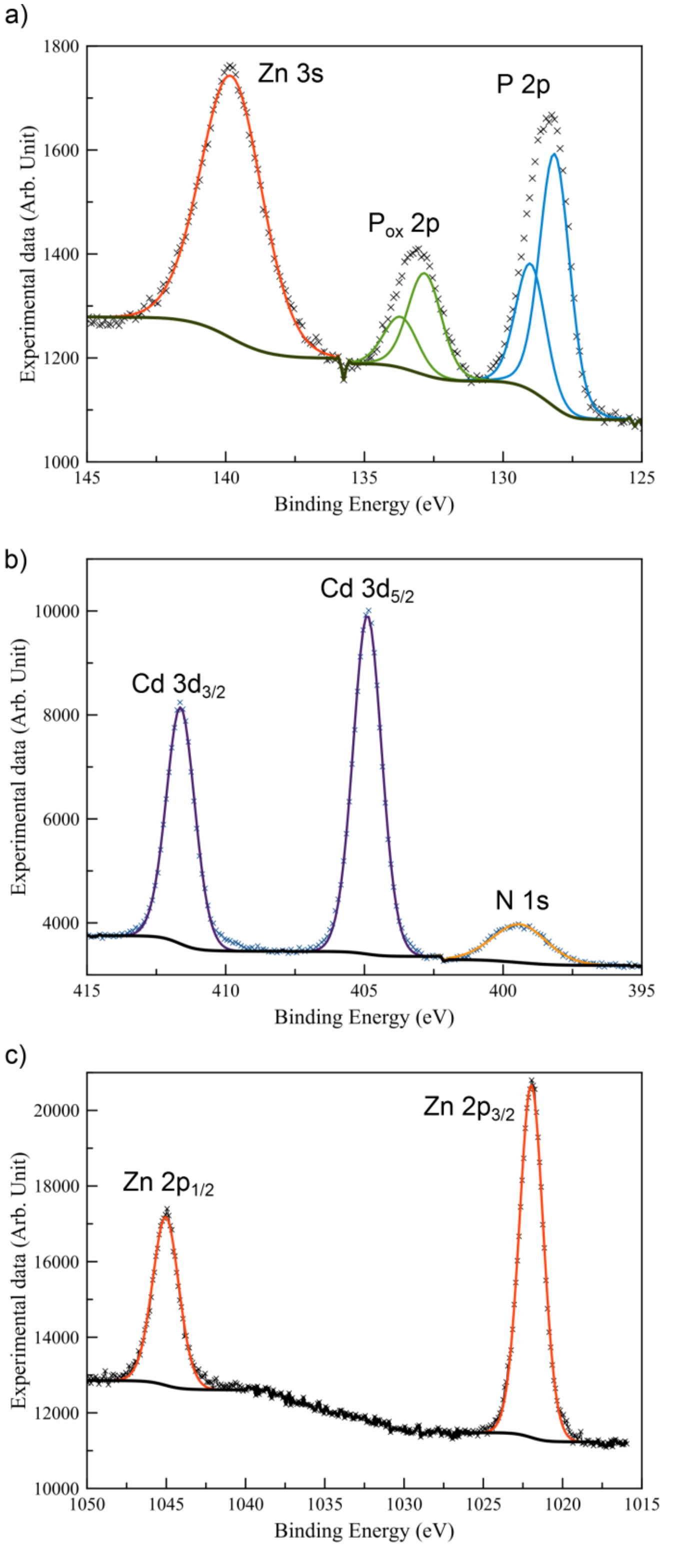

Supplement: Supplementary file 1 [file nanomaterials-12-03364-s001.zip › nanomaterials-1852768-supplementary.pdf]
